# Case Report: Practical approach to unmask unspecific adverse effects under lipid-lowering medication

**DOI:** 10.3389/fcvm.2025.1604129

**Published:** 2025-05-27

**Authors:** Thomas Büttner, Gunther Hartmann, Martin Coenen

**Affiliations:** Institute of Clinical Chemistry and Clinical Pharmacology, University Hospital Bonn, Bonn, Germany

**Keywords:** nocebo, intolerance, statins, provocation, case report

## Abstract

The nocebo effect, driven by negative expectations rather than pharmacological mechanisms, contributes significantly to medication non-adherence, particularly in lipid-lowering therapy. Up to 50% of reported statin-related adverse effects may result from nocebo responses, leading to unnecessary discontinuation and increased cardiovascular risk. Blinded provocation tests may offer a solution for the differentiation of true drug intolerance from nocebo-driven symptoms. Although this methodology is well-established in experimental studies, it has not been transferred to routine clinical practice so far. We present a 65-year-old female with hypercholesterolemia and cardiovascular risk factors who experienced recurrent, dose-dependent left-sided lower abdominal pain with different lipid-lowering drugs. These symptoms prompted repeated and ultimately continuous treatment discontinuations, each followed by resolution of complaints. Despite extensive evaluations, no organic cause was found. To assess the role of nocebo effects, a six-week single-blinded, placebo-controlled crossover provocation test with a commercially available placebo preparation and atorvastatin placed in neutral pill containers was conducted. Upon initiation of the provocation phase, the patient experienced similar intermittent symptoms under both treatments. The pain ratings on a numeric rating scale did not significantly differ during placebo (mean: 2.75) and atorvastatin administration (mean: 3.26), suggesting that these symptoms were not pharmacologically induced. Following information of the patient, atorvastatin therapy could be continued. During continued intake over several weeks, symptoms further diminished, reinforcing the therapeutic value of addressing nocebo effects. This case report provides for the first time the structured and detailed step-by-step description of a pragmatic approach for a prospective blinded, placebo-controlled provocation testing that can directly be implemented in routine clinical practice. This method enables the distinction of true drug intolerance from nocebo effects, thereby enabling necessary therapies and highlighting its diagnostic and therapeutic potential.

## Introduction

Research indicates that a relevant proportion of reported adverse drug reactions of widely used medications result from nocebo effects, describing negative outcomes to medical treatments in clinical trials or clinical practice that cannot be attributed to the drug's genuine pharmacological action but rather result from negative expectations. These effects present a major challenge in medical care, often leading to reduced adherence and the premature discontinuation of necessary therapies. This highlights the importance of developing targeted strategies to identify and minimize such effects (1). Despite extensive evidence supporting the cardiovascular benefits of statins, a large proportion of high-risk patients fail to achieve guideline-recommended low-density lipoprotein cholesterol (LDL-C) levels (2). One reason is the frequently reported statin intolerance, with many patients discontinuing therapy due to perceived adverse effects. However, studies indicate that up to 50% of statin-related adverse effects may be attributable to the nocebo effect (3).

There remains an unmet clinical need for strategies to differentiate true drug intolerance in general, and particularly to statins, from nocebo-driven adverse effects, thereby enabling the continuation of essential therapies. Blinded provocation tests may offer a solution for individual patients in the clinical practice (4, 5). Surprisingly, although this methodology is well-established in experimental studies investigating adverse effects of statins (6–8), it has not been transferred to routine clinical diagnostics so far.

This case report presents for the first time the structured and detailed step-by-step description of a protocol for a prospective, single-blinded, placebo-controlled provocation test in routine clinical practice in a patient with suspected statin intolerance to unmask adverse effects as unspecific, demonstrating its diagnostic and therapeutic potential.

## Materials and methods

### Case description

A 65-year-old female patient with hypercholesterolemia (LDL-C > 300 mg/dl) and a history of cardiovascular risk factors, including hypertension and a family history of early cardiovascular mortality, was referred to our Clinical Pharmacology Outpatient Department for further evaluation of suspected statin intolerance. Initial treatment with simvastatin had been well tolerated, but later attempts to escalate lipid-lowering therapy with ezetimibe and multiple statins (atorvastatin, rosuvastatin, pravastatin, and fluvastatin) led to recurrent, left-sided lower abdominal pain, which she had not previously experienced, within two weeks after initiation of therapy. The patient had no known allergies or food intolerances and had an omnivorous and varied diet. She reported a clear temporal relationship between medication intake and symptom onset, even in a dose-dependent manner as fluvastatin 20 mg was moderately tolerated, while 40 mg resulted in more severe symptoms. As a result, these complaints had repeatedly led to therapy discontinuation, and the symptoms improved after each interruption.

Comprehensive gastroenterological and gynecological investigations no definitive organic cause. Apart from slight meteorism in the colon an abdominal MRI revealed no abnormal findings, a colonoscopy ruled out tumors or chronic inflammatory bowel disease, and the gynecological findings were unremarkable. The laboratory results from blood, urine and stool cultures showed no abnormalities results apart from the known hypercholesterolemia. The patient discontinued statin therapy despite a high cardiovascular risk and resorted to various alternative lipid-lowering supplements of unproven efficacy, including red yeast rice, omega-3 fatty acids, coenzyme Q10, and vitamin B complexes, which failed to achieve LDL-C target levels.

### Diagnostics

The physical examination revealed no significant findings. The patient reported not feeling anxious, stressed, or depressed and denied the tendency of particular self-observation. Laboratory tests showed markedly elevated total cholesterol and LDL-C levels (356 and 277 mg/dl, respectively), while triglycerides were only slightly elevated (157 mg/dl). The *Naranjo Adverse Drug Reaction Probability Scale* (9) showed a score of 6 indicating a probable causal association with drug exposure. However, the repeated symptom onset upon introduction of several different lipid-lowering agents and resolution upon cessation was suggestive of a likely nocebo component unrelated to the pharmacological properties of these drugs. The patient was informed about the significance and mechanisms of the nocebo effect using a positive language, and it was discussed with her that she might also be experiencing this effect. Given the absence of confirmatory diagnostic markers, an evaluation strategy was necessary to objectively determine the cause of the intolerance.

### Intervention

**Figure 1:**
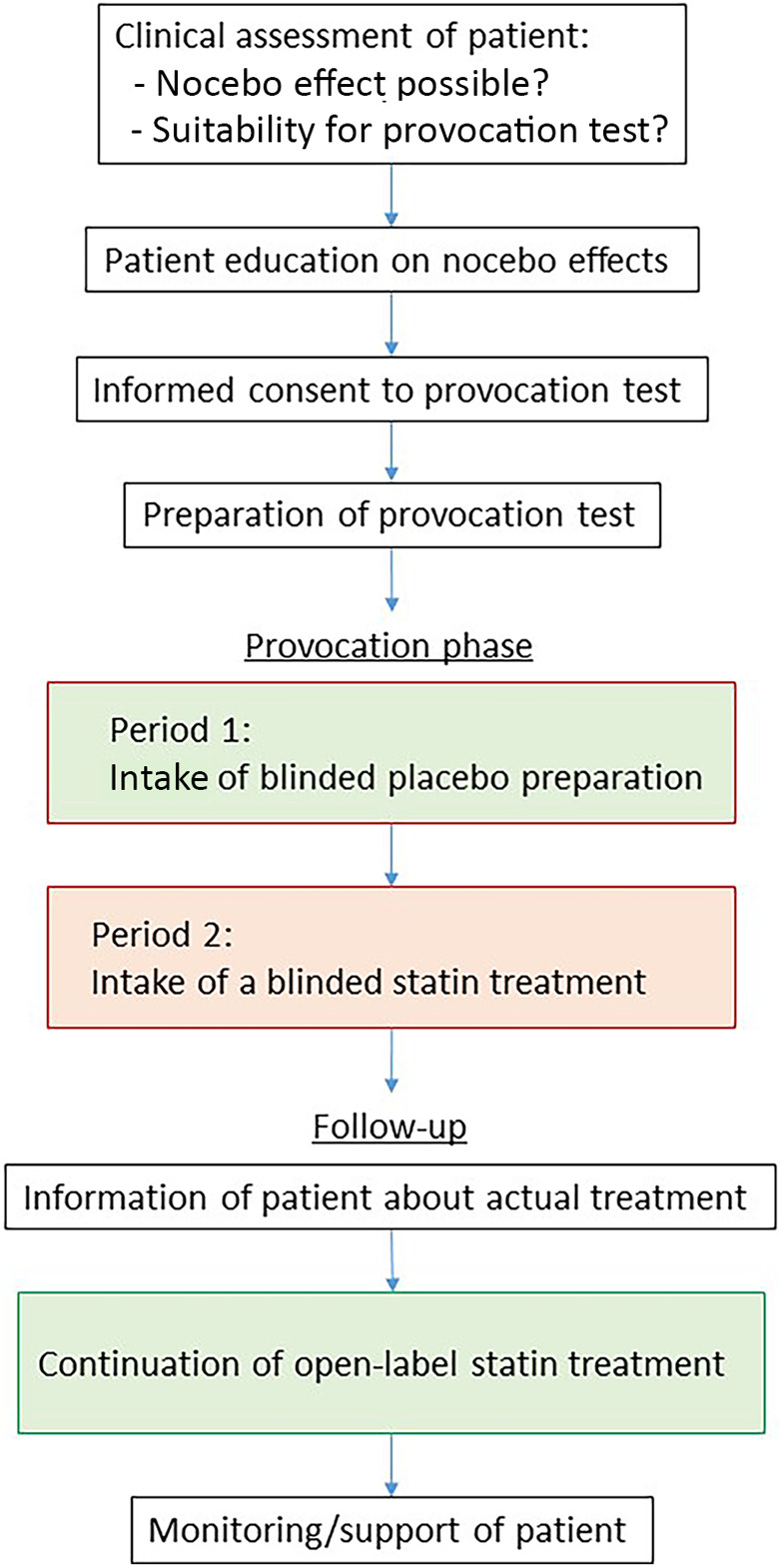
Schematic illustration of the process of the blinded provocation test including preparation and follow-up.

To delineate a potential nocebo effect, a single-blinded two-period crossover provocation test with placebo and atorvastatin (Atorvastatin AL 40 mg, Aliud Pharma, Laichingen, Germany) over six weeks (three weeks each) was conducted (Figure 1). Atorvastatin was chosen as the active ingredient as the patient had previously reported intolerance to it, and because a generic product was available in the form of a white, unmarked tablet with a neutral appearance, suitable for a pragmatic blinding. Furthermore, a matching commercially available placebo preparation (P-Tabletten weiß 7 mm, Zentiva, Prague, Czech Republic) was used. Both were placed in neutral pill containers to ensure patient blinding. Prior to the provocation test, the patient consented to the procedure after being informed in detail about the benefits and risks.

The patient was instructed to take the tablets once daily, independently of meals, and to avoid any other medications or supplements that could confound the results. Throughout the test procedure, the patient was closely monitored and documented daily pain intensity in a symptom diary on a Numeric Rating Scale (NRS), stool characteristics, and any other perceived side effects. The outcome assessment was based on a comparison of the mean NRS scores documented during the two periods including calculation of standard deviations and statistical testing using an unpaired t-test to determine differences between the placebo and atorvastatin phases.

For practical reasons, the provocation was carried out in a single-blinded manner and the patient was not actually randomized, although she was led to believe otherwise. Instead, the scheme (Figure 1) already predetermined that she would initially receive the placebo first. This approach enabled the evaluation of non-specific reactions without pharmacological causes at first and additionally eliminated the need for a washout phase before verum application. The approach is outlined by a step-by-step procedure in Table 1.

**Table 1:** Step-by-step process of a blinded provocation testing protocol with clarifying comments (right side) to facilitate reproducibility in similar outpatient settings.

Step	Comments
1. Clinical assessment of the patient	• Identification of existing adverse effects • Evaluation for non-specific effects (e. g. reaction to several drugs with different mode of actions or safety profile) • Consideration of patient characteristics (e. g., anxiety, depression, heightened self-observation, expectations aligning with reported symptoms)
2. Patient education on nocebo effects using a positive language	
3. Obtaining informed consent for the provocation test by the patient	
4. Selection of active treatment	• Choice of compound and formulation which was not tolerated before • Unlabelled product, closely resembling the used placebo preparation in appearance
5. Selection of placebo:	• Consideration of manufacturing feasibility constraints • For practical reasons selection of a suitable placebo matching the active treatment as closely as possible • Preference for the use of a commercially available product for practical reasons
6. Selection of neutral packaging	
7. Determination of duration of provocation test	• Ensuring sufficient time for a measurable response
8. Selection of outcome measures and time points	• e. g. symptom assessment via Visual Analog Scale (VAS) • Planning of laboratory assessments where appropriate
9. Procurement of active treatment and placebo	• Order from a pharmacy or prescription to the patient asking to bring product to the next visit
10. Filling of product into bottles and labelling with a clear identification option	
11. Initiation of the provocation test	• Administration or dispensing of the blinded placebo preparation to the patient
12. End of the placebo treatment and start of the blinded treatment with active compound	
13. Completion of the provocation test	
14. Analysis of outcome parameters and conclusion on the likelihood of nocebo effects	
15. Information of the patient	• Explanation of the provocation test and disclose of the actual treatments
16. Joint decision with the patient on the continuation of active treatment	
17. Follow-up with support and monitoring of patient	

## Results

**Figure 2:**
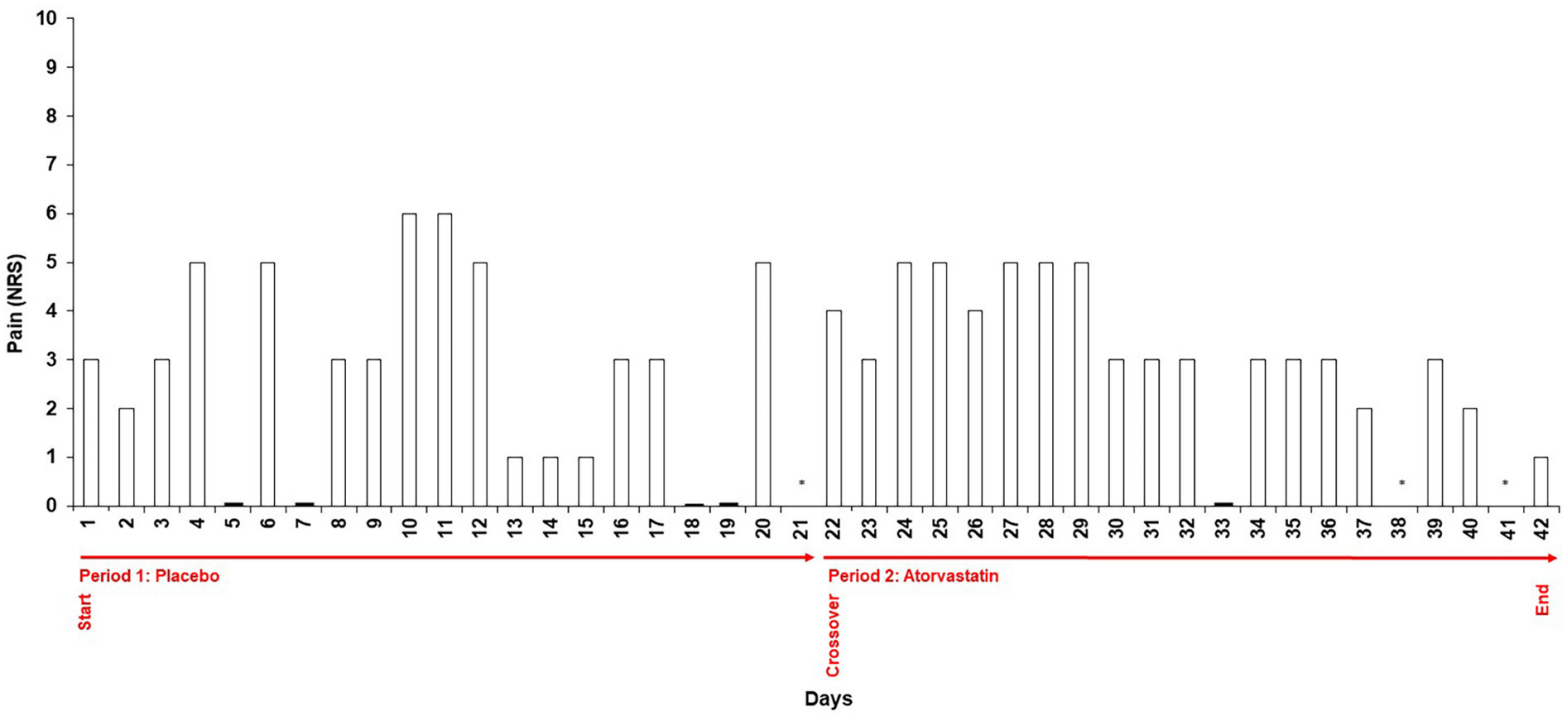
Daily symptom intensity ratings during blinded provocation testing. Numeric Rating Scale (NRS) scores (0–10) recorded daily by the patient throughout the two 21-day treatment periods. Period 1 represents the placebo phase; Period 2 represents the atorvastatin phase. Symptom intensity of abdominal discomfort was documented in a structured diary. The figure illustrates the variability of symptom reporting across both conditions, with comparable ratings observed during placebo and statin intake. Days without reported NRS scores are marked with N/A.

The patient was symptom-free before the start of the provocation test. During the three weeks of blinded placebo administration, the patient reported intermittent abdominal discomfort. Symptoms were sporadic and not consistently linked to medication intake. At the crossover point, the patient switched to the alternate treatment (blinded atorvastatin intake). Symptom fluctuations persisted without a clear association with statin administration. The intensity of symptoms reported during the placebo phase corresponded to that reported during the statin phase (Figure 2). The patient described the symptoms as mild, with a median intensity of 3/10 on the NRS which she subjectively perceived as less severe than her previous experiences with lipid-lowering therapies (estimated as 6/10 on the NRS) although she reported five days with a symptom severity ≥5 during the placebo period and six days under atorvastatin intake. Mean NRS rating were 2.75 [standard deviation (SD) = 2.09] under placebo and 3.26 (SD: 1.41) under atorvastatin (Table 2). Differences between the two periods were not statistically significant (*p* = 0.263). Notably, the patient had assumed that the statin phase had actually been the placebo phase from her own perception. Stool characteristics were unremarkable and other side effects were not reported. Total and LDL-Cholesterol levels showed a marked reduction to 211 mg/dl and 129 mg/dl respectively under atorvastatin therapy, highlighting the clinical importance of therapy resumption (Table 2).

**Table 2:** Symptom ratings and cholesterol levels during placebo and atorvastatin administration.

Parameter	Baseline	After 3 weeks of blinded placebo	After 3 weeks of blinded atorvastatin
Symptom intensity (NRS)	2/10		
Median symptom intensity (NRS)		3/10	3/10
Mean symptom intensity (SD)		2.75 (2.09)	3.26 (1.41)
Cholesterol (mg/dl)	356	371	211
LDL-C (mg/dl)	277	285	129

Overview of daily symptom ratings documented by the patient using a Numeric Rating Scale (NRS, 0–10) at baseline and during - and laboratory findings of cholesterol and LDL-cholesterol after - the blinded placebo and atorvastatin phases. The table presents median and mean values and standard deviations of NRS ratings in each period.

LDL-C, low-density lipoprotein cholesterol; NRS, Numeric Rating Scale; SD, standard deviation.

### Patient perspective

By the end of the test procedure, the patient expressed surprise at the results and acknowledged that symptoms had not worsened substantially under statin treatment. Given these findings, the patient agreed to resume statin therapy under continued medical supervision, with a focus on monitoring for any clinically significant adverse effects rather than perceived intolerance. During a short follow-up phase, the patient's symptoms decreased even further, supporting the conclusion that the complaints were caused by nonspecific effects. Ultimately, the patient's concerns regarding side effects were alleviated, and her overall well-being was significantly improved.

## Discussion

The definition of the clinical syndrome of statin intolerance of several guidances by working groups and professional societies simply relies on the clinical assessment of adverse effects and laboratory tests that occurred during administration of at least two different statins (10, 11). Overall, the findings in our patient strongly indicate that the symptoms were driven by nocebo effects rather than a true pharmacological intolerance. The structured monitoring of gastrointestinal symptoms throughout the provocation phase revealed that the patient experienced fluctuations in symptom intensity irrespective of statin exposure although the mean symptom rating differed slightly. However, statistical analyses, while essential for the systematic evaluation of clinical trials, are not appropriate for interpreting this individual case and can only serve to illustrate the effect size. The key observation is that symptoms similar to those reported under atorvastatin intake occurred under placebo at all. Therefore, the clinical diagnosis of statin intolerance actually was not appropriate and could easily be unmasked by a blinded rechallenge simply as the result of nocebo effects enabling the continuation of the important evidence-based lipid-lowering therapy to reduce the risk of cardiovascular patients (12).

Our findings align with large-scale n-of-1 trials, such as the SAMSON and StatinWISE trials which demonstrated that patients who previously reported statin-associated symptoms frequently experience similar adverse effects under placebo conditions during structured provocation testing (6, 7). The majority of patients were able to successfully resume statin therapy (6, 8). Our case further supports existing evidence indicating that subjective symptom perception is strongly influenced by patient expectations, prior experiences, and external factors unrelated to the pharmacological action of the drug (3). Presumably due to an earlier medical attribution of her – potentially coincidental - symptoms as “side effects” our patient developed a perceived causal link that likely contributed to the persistence of a nocebo effect. The patient denied having searched for symptoms in the package leaflet herself or having any relatives with similar complaints. However, she mentioned she had overheard talks claiming pronounced toxicity of statins in public. No signs of heightened anxiety, depression, or excessive self-monitoring—factors commonly associated with the development of nocebo effects (13–16) were identified.

Unlike previous clinical studies with larger patient cohorts, this is, to our knowledge, the first report providing a structured and detailed, individualized assessment using a blinded, placebo-controlled provocation test for diagnostic purposes in routine clinical practice rather than in a research context. It describes the step-by-step procedure and demonstrates its feasibility and applicability in real-world clinical settings (Table 1). To warrant a pragmatic approach that can be directly implemented in routine clinical practice, the patient received approved drug formulations in a single-blinded manner without actual randomization. Generally, an open-label placebo run-in phase could serve as a negative control. However, in the present case we opted against this approach because of the required long treatment periods and the short-term need of an effective lipid-lowering therapy for the patient. Considering the high costs and limited availability of alternative lipid-lowering agents, such as proprotein convertase subtilisin/kexin type 9 (PCSK9) inhibitors, verifying intolerance through a blinded rechallenge before escalating therapy represents a rational approach.

Strategic communication is a key factor in mitigating nocebo effects. The way potential side effects are framed by clinicians has a profound impact on patient expectations and subsequent symptom perception (17, 18). In line with international guidelines for dealing with nocebo effects (19) we educated the patient about non-specific drug effects using positive language emphasizing the high prevalence of nocebo effects in lipid-lowering therapy. This helped the patient to understand that perceived adverse effects may not be directly related to the medication itself. Furthermore, providing patients with personalized risk-benefit analyses can significantly enhance adherence, as they gain a clearer understanding of the necessity and advantages of continuing therapy. Structured educational interventions and shared decision-making models should therefore be integrated into routine clinical practice to empower patients to make informed choices based on clinical evidence rather than expectations (17, 18). Providing information about the provocation procedure is also a fundamental prerequisite for conducting such tests. In our case, this alone may have contributed to a reduction of symptoms under both, the active treatment and placebo conditions. However, it might also have triggered symptoms occurring in both phases (6). In either case, nonspecific effects can be considered as having been uncovered.

The case shows that this approach is not only diagnostically valuable but also therapeutically beneficial, as it allowed the patient to regain confidence in her prescribed therapy facilitating successful therapy resumption, ultimately achieving substantial LDL-C reduction and cardiovascular risk mitigation. The results underscore the utility of blinded provocation testing in differentiating true pharmacological adverse effects from nocebo-driven symptoms in patients intolerant to required medication that can be rolled out into the broader clinical application (Table 1). Of note, provocation testing using a placebo and interrupting drug therapy is only appropriate for patients who are receptive open to this kind of evaluation, who provide informed consent and for whom treatment can be safely paused for a limited period of time. Further research should explore scalable approaches to integrate nocebo management strategies into routine drug therapy to maximize treatment adherence and clinical benefits (4, 5, 20). A systematic approach should include the implementation of standardized protocols for blinded provocation testing to assess true statin intolerance into clinical practice to reduce unnecessary transitions to expensive second-line therapies, such as PCSK9 inhibitors as lipid-lowering alternatives, ultimately improving cardiovascular outcomes in high-risk patients.

## Data Availability

The data analyzed in this study is subject to the following licenses/restrictions: Data protection concerns. Requests to access these datasets should be directed to martin.coenen@ukbonn.de.
